# Body Mass Index and Mortality From All Causes and Major Causes in Japanese: Results of a Pooled Analysis of 7 Large-Scale Cohort Studies

**DOI:** 10.2188/jea.JE20100180

**Published:** 2011-11-05

**Authors:** Shizuka Sasazuki, Manami Inoue, Ichiro Tsuji, Yumi Sugawara, Akiko Tamakoshi, Keitaro Matsuo, Kenji Wakai, Chisato Nagata, Keitaro Tanaka, Tetsuya Mizoue, Shoichiro Tsugane

**Affiliations:** 1 Epidemiology and Prevention Division, Research Center for Cancer Prevention and Screening, National Cancer Center, Tokyo, Japan; 2 Division of Epidemiology, Department of Public Health and Forensic Medicine, Tohoku University Graduate School of Medicine, Sendai, Japan; 3 Department of Public Health, Aichi Medical University School of Medicine, Aichi, Japan; 4 Division of Epidemiology and Prevention, Aichi Cancer Center Research Institute, Nagoya, Japan; 5 Department of Preventive Medicine, Nagoya University Graduate School of Medicine, Nagoya, Japan; 6 Department of Epidemiology & Preventive Medicine, Gifu University School of Medicine, Gifu, Japan; 7 Department of Preventive Medicine, Saga Medical School, Faculty of Medicine, Saga University, Saga, Japan; 8 Department of Epidemiology and International Health, Research Institute National Center for Global Health and Medicine, Tokyo, Japan

**Keywords:** body mass index, mortality, cancer, heart disease, cerebrovascular disease

## Abstract

**Background:**

We pooled data from 7 ongoing cohorts in Japan involving 353 422 adults (162 092 men and 191 330 women) to quantify the effect of body mass index (BMI) on total and cause-specific (cancer, heart disease, and cerebrovascular disease) mortality and identify optimal BMI ranges for middle-aged and elderly Japanese.

**Methods:**

During a mean follow-up of 12.5 years, 41 260 deaths occurred. The Cox proportional hazards model was used to estimate hazard ratios (HRs) for each BMI category, after controlling for age, area of residence, smoking, drinking, history of hypertension, diabetes, and physical activity in each study. A random-effects model was used to obtain summary measures.

**Results:**

A reverse-J pattern was seen for all-cause and cancer mortality (elevated risk only for high BMI in women) and a U- or J-shaped association was seen for heart disease and cerebrovascular disease mortality. For total mortality, as compared with a BMI of 23 to 25, the HR was 1.78 for 14 to 19, 1.27 for 19 to 21, 1.11 for 21 to 23, and 1.36 for 30 to 40 in men, and 1.61 for 14 to 19, 1.17 for 19 to 21, 1.08 for 27 to 30, and 1.37 for 30 to 40 in women. High BMI (≥27) accounted for 0.9% and 1.5% of total mortality in men and women, respectively.

**Conclusions:**

The lowest risk of total mortality and mortality from major causes of disease was observed for a BMI of 21 to 27 kg/m^2^ in middle-aged and elderly Japanese.

## INTRODUCTION

Obesity is responsible for a serious health burden because of its association with type 2 diabetes mellitus, cardiovascular diseases, and some types of cancer.^1^ As a measure of relative body weight, body mass index (BMI) is an easy-to-obtain, acceptable proxy for thinness and fatness, and has been found to be directly related to health risks and death rates in many populations. According to the World Health Organization (WHO), the currently recommended BMI cut-off points for overweight and obesity are 25 kg/m^2^ or greater and 30 kg/m^2^ or greater, respectively.

Although these criteria were intended for international use, debate has centered on using the same cut-off points for Asian populations because of the high prevalence in those populations of type 2 diabetes mellitus and cardiovascular disease risk factors in individuals with a BMI less than 25 kg/m^2^, as well as differences in the relationships between BMI, body fat percentage, and body fat distribution.^2^ In 2002, a WHO expert consultation addressed this issue and concluded that there were no clear cut-off points for overweight and obesity in Asians. Based on international classifications, the consultation defined a BMI cut-off point of 23 kg/m^2^ or greater as “increased risk” and a cut-off point of greater than 27.5 kg/m^2^ as “high risk”.^3^ However, in a recent, large pooled analysis of more than 1.1 million Asians, different patterns of association were observed between East Asians (Chinese, Japanese, and Koreans) and other Asians (Indians and Bangladeshis).^4^ Among East Asians, the lowest risk of death was seen among those with a BMI of 22.6 to 27.5, and the risk was elevated among those with a BMI higher or lower than that range. In the cohorts comprising Indians and Bangladeshis, the risk of death was increased for a BMI of 20.0 or less as compared with those with a BMI of 22.6 to 25.0, and there was no increase in risk associated with a high BMI. Considering the variation just within Asia, country-specific BMI cut-off points should be developed for public health interventions.

To date, many prospective cohort studies have evaluated the association between BMI and mortality in the Japanese population^5^^–^^10^; some showed a U-shaped^7^^,^^9^ or reverse J-shaped association,^10^ but others did not.^5^^,^^6^^,^^8^ These studies defined BMI categories differently and controlled for different confounding variables. In the present study, we pooled 7 cohort studies in Japan to clarify the role of relative body weight on total mortality and major causes of mortality (cancer, heart disease, and cerebrovascular disease) in the Japanese population. In the present analysis of more than 350 000 subjects we also aimed to identify an optimal BMI range for middle-aged and elderly Japanese.

## METHODS

### Study population

In 2006, the Research Group for the Development and Evaluation of Cancer Prevention Strategies in Japan initiated a pooling project using original data from major cohort studies to evaluate the association between lifestyle and major forms of cancer and mortality in Japanese. Topics for the pooled analysis were determined on the basis of discussions among all authors and were evaluated with respect to their scientific and public health importance.^11^^,^^12^ To maintain the quality and comparability of data, we established a priori inclusion criteria: namely, population-based cohort studies that (1) were conducted in Japan and started in the mid-1980s to mid-1990s, (2) included more than 30 000 participants, (3) obtained information on BMI calculated by height and weight reported in a validated questionnaire at baseline, and (4) collected any cause of mortality during the follow-up period. Seven ongoing studies that met these criteria were identified: the Japan Public Health Center-based Prospective Study, Cohort I (JPHC-I)^13^; the Japan Public Health Center-Based Prospective Study, Cohort II (JPHC-II)^13^; the Japan Collaborative Cohort Study (JACC)^14^; the Miyagi Cohort Study (MIYAGI)^15^; the Ohsaki National Health Insurance Cohort Study (OHSAKI)^16^; the Three-Prefecture Aichi (3-pref AICHI)^17^; and the Takayama Study (TAKAYAMA).^18^ When analyzing individual results of each study, subjects with a previous history of any cancer, stroke, or myocardial infarction or with missing or implausible data (BMI <14 or ≥40) on BMI were excluded. Table 1 profiles the studies included in the analyses. Each study was approved by the appropriate institutional review board.

**Table 1.** Characteristics of the 7 cohort studies included in a pooled analysis of body mass index and risk of all-cause and major-cause mortality

Study	Population	Age (years) at baseline survey	Year(s) of baseline survey	Population size	Rate of response (%) to baseline questionnaire	Method of follow-up	The present pooled analysis						

							Age (years)	Last follow-up time	Mean duration of follow-up (years)	Size of cohort		Number of total deaths	
	
										Men	Women	Men	Women
JPHC-I	Japanese residents of 5 public health center areas in Japan	40–59	1990	61 595	82%	Death certificates	40–59	2005	14.2	23 156	26 104	2392	1194

JPHC-II	Japanese residents of 6 public health center areas in Japan	40–69	1993–1994	78 825	80%	Death certificates	40–69	2005	11.3	29 015	32 484	3672	1802

JACC	Residents of 45 areas throughout Japan	40–79	1988–1990	110 792	83%	Death certificates	40–79	2006	14.7	41 639	57 147	10 575	7351

MIYAGI	Residents of 14 municipalities in Miyagi Prefecture, Japan	40–64	1990	47 605	92%	Death certificates	40–64	2004 (all causes),	13.5	20 832	22 616	2097	1041
								2001 (cause-specific)	10.3			1409	699

OHSAKI	Residents of 14 municipalities in Miyagi Prefecture, Japan	40–79	1994	52 029	95%	Death certificates	40–79	2006	10.0	21 008	22 886	3675	2015

3-pref AICHI	Residents of 2 municipalities in Aichi Prefecture, Japan	40–103	1985	33 529	90%	Death certificates	40–103	2000	11.7	13 841	15 296	2516	1866

TAKAYAMA	Japanese residents of Takayama, Gifu, Japan	≥35	1992	31 552	85%	Death certificates	35–101	1999	6.9	12 601	14 797	1017	767

Total										162 092	191 330	25 944	16 036

Abbreviations: JPHC, Japan Public Health Center-based prospective Study; JACC, The Japan Collaborative Cohort Study; MIYAGI, The Miyagi Cohort Study; OHSAKI, Ohsaki National Health Insurance Cohort Study; 3-pref AICHI, The Three Prefecture Study - Aichi portion; TAKAYAMA, Takayama Study.

### Follow-up and outcome ascertainment

Subjects were followed from the baseline survey (JPHC-I, 1990; JPHC-II, 1993–1994; JACC, 1988–1990; MIYAGI, 1990; OHSAKI, 1994; 3-pref AICHI, 1985; TAKAYAMA, 1992) to the last date of follow-up for any cause of mortality (JPHC-I, 2005; JPHC-II, 2005; JACC, 2006; MIYAGI, 2004 [2001 for cause-specific mortality]; OHSAKI, 2006; 3-pref AICHI, 2000; TAKAYAMA, 1999) in each study. Residence status, including survival, was confirmed through the residential registry.

Information on cause of death was obtained from death certificates provided by the Ministry of Health, Labour and Welfare with the permission of the Ministry of Internal Affairs and Communications. Cause of death was defined according to the International Classification of Disease, 10th version (ICD-10).^19^ Resident and death registration are required by law in Japan. The outcome of the present study was defined as all-cause mortality, including the 3 major causes of death among Japanese, specifically, cancer (ICD-10: C00–C97), heart disease (ICD-10: I20–I52), and cerebrovascular disease (ICD-10: I60–I69).

### BMI assessment

Body weight and height were self-reported in the baseline questionnaire conducted at each study. BMI was calculated as weight divided by the square of the height (kg/m^2^). It was then divided into 7 categories using cut-off points that were identical among the studies, that is, 14 to 18.9, 19 to 20.9, 21 to 22.9, 23 to 24.9 (reference), 25 to 26.9, 27 to 29.9, and 30 to 39.9 kg/m^2^. The cut-off points were derived from a US study (<21, 21.0–22.9, 23.0–24.9, 25.0–26.9, 27.0–29.9, and ≥30.0 kg/m^2^) that enrolled a reasonably large number of subjects and carefully accounted for methodologic problems.^20^ Due to the large number of lean people, individuals with a BMI less than 21 kg/m^2^ were subdivided into 2 groups in the present analysis: 14.0 to 18.9 kg/m^2^ and 19 to 20.9 kg/m^2^. This decision was based on our observation in the JPHC study that both BMI extremes are important determinants of total mortality^9^ and cancer occurrence and mortality.^21^

### Statistical analysis

Time at risk was calculated as the duration from the date of the baseline survey in each study until the date of death or end of follow-up, whichever came first. In each study, sex-specific hazard ratios (HRs) and their 95% confidence intervals (CIs) were estimated for all-cause and cause-specific (cancer, heart disease, cerebrovascular disease, and other) mortality for each BMI category using the Cox proportional hazards model. Each study performed 2 types of adjustment for estimation of HRs: age (years, continuous) and area (JPHC-I, JPHC-II, and JACC only) (HR1). Further multivariate adjustments were conducted by including covariates in the model that were either known or suspected confounding factors, ie, cigarette smoking (for men: never smoker, past smoker, current smoker of 1 to 19 cigarettes/day or ≥20 cigarettes/day; for women: never smoker, past smoker, or current smoker), alcohol drinking (nondrinkers [never- and ex-drinker], occasional drinkers [less than once per week], regular drinkers [almost daily for OHSAKI and 3-pref AICHI; ≥5 days/week for JPHCI, JPHCII, and JACC; ≥5 times/week for MIYAGI; and ≥4 to 6 days/week for TAKAYAMA]), history of hypertension (no, yes), history of diabetes (no, yes), and leisure-time sports or physical exercise (less than almost daily, almost daily) (HR2). All included studies were population-based, and blood data were available for only a part of 1 study. We therefore used self-reported past history of diseases to control for hypertension and diabetes. We conducted an additional analysis that excluded deaths within 5 years from both the numerator and denominator (HR3).^22^^,^^23^ For men, we conducted stratified analysis by smoking status, namely, of never smokers and current smokers. An indicator term for missing data was created for each covariate.^24^ SAS (version 9.1; SAS Institute, Cary, NC, USA) and Stata (version 11; Stata Corporation, College Station, TX, USA) statistical software were used for these analyses.

A random-effects model was used to obtain summary measures of the HRs from the individual studies for each category. The study-specific HRs were weighted by the inverse of the sum of their variance and the estimated between-studies variance component. These values from the individual studies were then combined using a random-effects model. The impact of heterogeneity was measured by using the *I*^2^ statistic, which describes the proportion of total variation in study estimates that is due to heterogeneity. Although there is no universal rule to define mild, moderate, or severe heterogeneity, it is reasonable to assume that a value less than 30% represents mild heterogeneity and that a value greater than 50% represents substantial heterogeneity.^25^ Stata software was used for the meta-analysis.

In addition, to express the impact of BMI on the risk of mortality, the population-attributable fraction (PAF) was estimated and expressed as a percentage.^26^ Using HR2 and prevalence in each category, we calculated the PAF attributable to high BMI (≥27 kg/m^2^ for men and women), assuming subjects in these BMI categories moved to the reference category (23–25 kg/m^2^). The reference category was based on the BMI range in which total mortality was lowest for men and women, respectively. We applied this reference category to all end points, and when the HR was less than 1.0, the PAF was calculated as a minus value. This occurred in only 1 category: the PAF of cancer due to a BMI of 27 to 30 kg/m^2^ in men was −0.10%, and together with the PAF due to a BMI of 30 to 40 (0.29%), the PAF of cancer due to high BMI (≥27 kg/m^2^) was 0.2%.

## RESULTS

The present study included 353 422 adults (162 092 men and 191 330 women) from 7 ongoing large-scale, population-based, prospective studies in Japan (Table 1). During 4 399 108 person-years of follow-up (mean 12.5 years/person), 41 260 deaths were identified (25 944 men and 15 316 women), including 15 690 deaths from cancer (10 115 men and 5575 women), 5940 deaths from heart disease (3378 men and 2562 women), 5071 deaths from cerebrovascular disease (2820 men and 2251 women), and 14 451 deaths from other causes (8950 men and 5501 women). The baseline characteristics of the study subjects by BMI category have been previously published.^4^^,^^5^^,^^7^^,^^8^^,^^20^^,^^26^^–^^28^

Table 2 summarizes the results of pooled analyses of BMI and mortality in men. When the model was fully adjusted for confounding variables (HR2), a reverse J-shaped association was observed for mortality from all causes, cancer, and other causes. Regarding these outcomes, a statistically significant increased risk was observed for all 3 categories among individuals with a BMI less than 23. As compared with a BMI range of 23 to 25 kg/m^2^, the HRs for BMI ranges 14 to 19, 19 to 21, and 21 to 23 kg/m^2^ were 1.78, 1.27, and 1.11 for all-cause death, 1.44, 1.23, and 1.10 for cancer death, and 2.15, 1.42, and 1.17 for other-cause death, respectively. The HR continued to decrease even for a BMI greater than 25 kg/m^2^, and the BMI range 25 to 27 kg/m^2^ seemed to be the lowest risk group for these outcomes. Increased risk among individuals with a high BMI was limited to those with a BMI of 30 to 40 kg/m^2^ (obesity); the HR was 1.36 for all-cause death (statistically significant), 1.20 for cancer death (not statistically significant), and 1.29 for other-cause death (not statistically significant).

**Table 2.** Pooled analysis of BMI and mortality (Men)

		14–<19	19–<21	21–<23	23–<25	25–<27	27–<30	30–<40	Heterogeneity I squared (%) and *P* for the	
		HR (95% CI)	HR (95% CI)	HR (95% CI)	HR (95% CI)	HR (95% CI)	HR (95% CI)	HR (95% CI)	lowest category	highest category
*All Causes*										
Number of subjects	(*n* = 162 092)	9933	28 571	44 035	42 354	23 238	11 448	2513		
Person-years	1 967 103	108 482	342 361	538 369	522 805	287 923	141 921	25 243		
Number of deaths	(*n* = 25 944)	3162	5717	7022	5519	2728	1420	376		
Crude rate (per 100 000)		2914.77	1669.88	1304.31	1055.65	947.48	1000.56	1489.53		
Age-standardized rate (per 100 000)		2009.45	1483.13	1283.1	1144.92	1086.56	1205.09	1495.49		
Age- and area-adjusted (HR1)^a^		**1.83** **(1.64–2.05)**	**1.30** **(1.24–1.37)**	**1.12** **(1.05–1.19)**	1.00 (Reference)	0.95 (0.91–0.996)	1.09 (0.97–1.21)	**1.42** **(1.22–1.65)**	80.6% (*P* < 0.0001)	46.6% (*P* = 0.081)
Multivariate-adjusted (HR2)^b^		**1.78** **(1.60–1.98)**	**1.27** **(1.22–1.33)**	**1.11** **(1.04–1.18)**	1.00 (Reference)	0.94 (0.90–0.99)	1.07 (0.97–1.17)	**1.36** **(1.19–1.55)**	77.1% (*P* < 0.0001)	32.3% (*P* = 0.181)
Multivariate-adjusted, excl. ​ early death (HR3)^c^		**1.64** **(1.50–1.79)**	**1.24** **(1.19–1.29)**	**1.10** **(1.03–1.17)**	1.00 (Reference)	0.96 (0.91–1.01)	1.09 (0.97–1.22)	**1.35** **(1.11–1.65)**	52.8% (*P* = 0.048)	59.4% (*P* = 0.022)

*Cancer*										
Number of subjects	(*n* = 162 092)	9933	28 571	44 035	42 354	23 238	11 448	2513		
Person-years	1 909 493	106 697	333 333	521 589	504 796	277 359	136 168	29 551		
Number of deaths	(*n* = 10 115)	1022	2252	2873	2269	1056	516	127		
Crude rate (per 100 000)		957.85	675.60	550.82	449.49	380.73	378.94	429.76		
Age-standardized rate (per 100 000)		730.77	614.48	541.64	479.33	426.71	437.22	526.94		
Age- and area-adjusted (HR1)^a^		**1.52** **(1.31–1.77)**	**1.29** **(1.19–1.40)**	**1.13** **(1.04–1.22)**	1.00 (Reference)	0.90 (0.83–0.96)	0.97 (0.85–1.10)	1.18 (0.95–1.47)	68.9% (*P* = 0.004)	27.8% (*P* = 0.226)
Multivariate-adjusted (HR2)^b^		**1.44** **(1.24–1.67)**	**1.23** **(1.13–1.34)**	**1.10** **(1.02–1.19)**	1.00 (Reference)	0.90 (0.84–0.97)	0.98 (0.86–1.12)	1.20 (0.97–1.50)	67.7% (*P* = 0.005)	27.2% (*P* = 0.231)
Multivariate-adjusted, excl. ​ early death (HR3)^c^		**1.27** **(1.12–1.43)**	**1.17** **(1.09–1.26)**	1.08 (0.997–1.18)	1.00 (Reference)	0.95 (0.87–1.03)	1.02 (0.86–1.22)	**1.29** **(1.05–1.58)**	27.6% (*P* = 0.218)	0.0% (*P* = 0.460)

*Heart Disease*										
Number of subjects	(*n* = 162 092)	9933	28 571	44 035	42 354	23 238	11 448	2513		
Person-years	1 909 493	106 697	333 333	521 589	504 796	277 359	136 168	29 551		
Number of deaths	(*n* = 3378)	383	671	887	725	411	237	64		
Crude rate (per 100 000)		358.96	201.30	170.06	143.62	148.18	174.05	216.57		
Age-standardized rate (per 100 000)		231.78	176.19	167.33	157.75	170.83	215.61	276.87		
Age- and area-adjusted (HR1)^a^		**1.47** **(1.24–1.74)**	**1.11** **(1.00–1.24)**	1.05 (0.95–1.16)	1.00 (Reference)	1.05 (0.86–1.29)	1.37 (0.998–1.87)	**1.85** **(1.43–2.39)**	27.7% (*P* = 0.217)	0.0% (*P* = 0.711)
Multivariate-adjusted (HR2)^b^		**1.45** **(1.21–1.74)**	**1.11** **(1.00–1.24)**	1.05 (0.95–1.16)	1.00 (Reference)	1.03 (0.84–1.25)	1.28 (0.95–1.74)	**1.71** **(1.32–2.22)**	34.5% (*P* = 0.164)	0.0% (*P* = 0.765)
Multivariate-adjusted, excl. ​ early death (HR3)^c^		**1.28** **(1.04–1.59)**	1.10 (0.96–1.24)	1.01 (0.89–1.15)	1.00 (Reference)	1.04 (0.83–1.31)	1.17 (0.83–1.65)	**1.72** **(1.22–2.43)**	25.7% (*P* = 0.232)	13.4% (*P* = 0.328)

*Cerebrovascular Disease*										
Number of subjects	(*n* = 162 092)	9933	28 571	44 035	42 354	23 238	11 448	2513		
Person-years	1 909 493	106 697	333 333	521 589	504 796	277 359	136 168	29 551		
Number of deaths	(*n* = 2820)	332	625	737	605	309	162	50		
Crude rate (per 100 000)		311.16	187.50	141.30	119.85	111.41	118.97	169.20		
Age-standardized rate (per 100 000)		201.17	161.94	138.77	133.33	132.93	153.72	218.73		
Age- and area-adjusted (HR1)^a^		**1.43** **(1.20–1.71)**	**1.21** **(1.06–1.39)**	1.03 (0.92–1.15)	1.00 (Reference)	1.01 (0.88–1.16)	1.19 (0.996–1.41)	**1.81** **(1.35–2.42)**	22.5% (*P* = 0.257)	0.0% (*P* = 0.511)
Multivariate-adjusted (HR2)^b^		**1.53** **(1.26–1.85)**	**1.28** **(1.10–1.49)**	1.05 (0.94–1.17)	1.00 (Reference)	0.97 (0.84–1.11)	1.10 (0.92–1.31)	**1.64** **(1.23–2.20)**	29.4% (*P* = 0.204)	0.0% (*P* = 0.671)
Multivariate-adjusted, excl. ​ early death (HR3)^c^		**1.52** **(1.23–1.89)**	**1.21** **(1.06–1.38)**	1.02 (0.89–1.15)	1.00 (Reference)	0.95 (0.75–1.20)	1.11 (0.91–1.36)	**1.54** **(1.06–2.24)**	22.3% (*P* = 0.259)	4.9% (*P* = 0.391)

*Other Causes*										
Number of subjects	(*n* = 162 092)	9933	28 571	44 035	42 354	23 238	11 448	2513		
Person-years	1 909 493	106 697	333 333	521 589	504 796	277 359	136 168	29 551		
Number of deaths	(*n* = 8950)	1388	2047	2347	1751	861	448	108		
Crude rate (per 100 000)		1300.87	614.10	449.97	346.87	310.43	329.00	365.46		
Age-standardized rate (per 100 000)		853.68	538.23	443.38	380.67	361.97	362.07	370.2		
Age- and area-adjusted (HR1)^a^		**2.49** **(2.14–2.90)**	**1.43** **(1.33–1.55)**	**1.18** **(1.08–1.29)**	1.00 (Reference)	0.94 (0.85–1.04)	1.08 (0.97–1.20)	**1.35** **(1.00–1.83)**	70.9% (*P* = 0.002)	53.0% (*P* = 0.047)
Multivariate-adjusted (HR2)^b^		**2.15** **(2.10–2.79)**	**1.42** **(1.32–1.54)**	**1.17** **(1.07–1.28)**	1.00 (Reference)	0.93 (0.84–1.03)	1.05 (0.95–1.17)	1.29 (0.95–1.74)	65.6% (*P* = 0.008)	52.4% (*P* = 0.050)
Multivariate-adjusted, excl. ​ early death (HR3)^c^		**2.31** **(1.99–2.69)**	**1.43** **(1.30–1.57)**	**1.16** **(1.08–1.25)**	1.00 (Reference)	0.93 (0.84–1.02)	1.10 (0.98–1.24)	1.22 (0.85–1.76)	53.0% (*P* = 0.047)	51.2% (*P* = 0.056)

^a^Adjusted for age (years, continuous) and area (for JPHC-I, JPHC-II, and JACC only) (HR1).

^b^Further adjusted for cigarette smoking (never smoker, past smoker, current smoker of 1–19 cigarettes/day or ≥20 cigarettes/day), alcohol drinking (nondrinkers [never- or ex-drinker], occasional drinkers (less than once per week), regular drinkers (almost daily for OHSAKI and 3-pref AICHI; ≥5 days/week for JPHCI, JPHCII, and JACC; ≥5 times/week for MIYAGI; and ≥4–6 days/week for TAKAYAMA), history of hypertension (no, yes), history of diabetes (no, yes), and leisure-time sports or physical exercise (less than almost daily, almost daily) (HR2).

^c^Excluding deaths within 5 years (HR3). Bold text: *P* < 0.05.

For heart disease and cerebrovascular disease, a U-shaped or J-shaped association was observed. A statistically significant increased risk was observed for both the high and low BMI ranges. The HR was similar or slightly higher for a high BMI; the HRs for a BMI of 14 to 19, 19 to 21, and 30 to 40 kg/m^2^ were 1.45, 1.11, and 1.71 for heart disease and 1.53, 1.28, and 1.64 for cerebrovascular disease, respectively.

When subjects who died in the first 5 years of follow-up were excluded, most results were attenuated, but still significant (HR3). Through this process, the *I*^2^ for the lowest category improved, which suggests that different conditions of early death across studies were the main reason for the heterogeneity seen among individuals with a lower BMI. Due to the relatively small number of subjects in the highest BMI category, the same process increased the *I*^2^ in some outcomes for that category.

In women, a reverse J-shaped association was also observed for all-cause and other-cause mortality, but not for cancer (Table 3). For all-cause mortality, after fully adjusting for potential confounding factors (HR2) and using a BMI range of 23 to 25 kg/m^2^ as the basis for comparison, the HRs for BMI ranges 14 to 19, 19 to 21, 27 to 30, and 30 to 40 kg/m^2^ were estimated as 1.61, 1.17, 1.08, and 1.37, respectively. For cancer, a statistically significant increased risk was observed only for obesity, and there was no evidence of increased risk at any lower BMI range. After fully adjusting for confounding factors (HR2) and comparing with BMI range 23 to 25 kg/m^2^, the HR for BMI range 30 to 40 kg/m^2^ was 1.25. As with men, a U-shaped or J-shaped association was observed for heart disease and cerebrovascular disease in women. The risk elevation at lower and higher BMIs was more apparent for heart disease: the HRs for BMI ranges 14 to 19 and 30 to 40 kg/m^2^ were 1.77 and 1.79 for heart disease and 1.44 and 1.30 for cerebrovascular disease, respectively. For all-cause and other-cause mortality, exclusion of early deaths slightly attenuated the results, but they remained significant. Furthermore, heterogeneity seen in the lowest category became nonsignificant.

**Table 3.** Pooled analysis of BMI and mortality (Women)

		14–<19	19–<21	21–<23	23–<25	25–<27	27–<30	30–<40	Heterogeneity I squared (%) and *P* for the	
		HR (95% CI)	HR (95% CI)	HR (95% CI)	HR (95% CI)	HR (95% CI)	HR (95% CI)	HR (95% CI)	lowest category	highest category
*All Causes*										
Number of subjects	(*n* = 191 303)	15 027	34 289	50 450	44 316	26 341	16 066	4814		
Person-years	2 432 005	176 627	426 608	644 023	572 803	342 343	207 994	61 606		
Number of deaths	(*n* = 16 036)	2302	2929	3702	3155	2081	1341	526		
Crude rate (per 100 000)		1303.32	686.58	574.82	550.80	607.87	644.73	853.81		
Age-standardized rate (per 100 000)		941.03	671.12	602.87	587.28	623.57	662.05	861.61		
Age- and area-adjusted (HR1)^a^		**1.57** **(1.49–1.66)**	**1.15** **(1.08–1.22)**	1.03 (0.97–1.09)	1.00 (Reference)	1.06 (0.997–1.13)	**1.15** **(1.08–1.23)**	**1.51** **(1.37–1.65)**	0.0% (*P* = 0.436)	0.0% (*P* = 0.739)
Multivariate-adjusted (HR2)^b^		**1.61** **(1.53–1.71)**	**1.17** **(1.11–1.23)**	1.03 (0.98–1.09)	1.00 (Reference)	1.04 (0.98–1.10)	**1.08** **(1.02–1.16)**	**1.37** **(1.24–1.50)**	0.0% (*P* = 0.728)	0.0% (*P* = 0.800)
Multivariate-adjusted, excl. ​ early death (HR3)^c^		**1.55** **(1.45–1.65)**	**1.17** **(1.10–1.24)**	1.03 (0.98–1.09)	1.00 (Reference)	1.07 (0.95–1.21)	**1.10** **(1.03–1.18)**	**1.34** **(1.17–1.54)**	0.0% (*P* = 0.643)	50.5% (*P* = 0.059)

*Cancer*										
Number of subjects	(*n* = 191 303)	15 027	34 289	50 450	44 316	26 341	16 066	4814		
Person-years	2 359 991	173 647	416 348	626 108	554 620	329 853	200 022	59 393		
Number of deaths	(*n* = 5575)	554	970	1352	1244	789	491	175		
Crude rate (per 100 000)		319.04	232.98	215.94	224.30	239.20	245.47	294.65		
Age-standardized rate (per 100 000)		267.45	235.79	223.67	231.51	237.09	241.52	289.2		
Age- and area-adjusted (HR1)^a^		1.13 (0.95–1.35)	1.01 (0.92–1.10)	1.01 (0.90–1.13)	1.00 (Reference)	1.04 (0.95–1.13)	1.07 (0.96–1.19)	**1.30** **(1.11–1.52)**	56.8% (*P* = 0.031)	0.0% (*P* = 0.909)
Multivariate-adjusted (HR2)^b^		1.12 (0.93–1.35)	1.00 (0.92–1.09)	1.00 (0.90–1.12)	1.00 (Reference)	1.03 (0.94–1.13)	1.05 (0.94–1.17)	**1.25** **(1.07–1.47)**	59.1% (*P* = 0.023)	0.0% (*P* = 0.935)
Multivariate-adjusted, excl. ​ early death (HR3)^c^		1.10 (0.92–1.31)	1.00 (0.91–1.11)	1.00 (0.88–1.13)	1.00 (Reference)	1.04 (0.93–1.15)	1.11 (0.98–1.25)	**1.28** **(1.07–1.54)**	36.1% (*P* = 0.153)	0.0% (*P* = 0.988)

*Heart Disease*										
Number of subjects	(*n* = 191 303)	15 027	34 289	50 450	44 316	26 341	16 066	4814		
Person-years	2 359 991	173 647	416 348	626 108	554 620	329 853	200 022	59 393		
Number of deaths	(*n* = 2562)	385	429	548	423	300	381	96		
Crude rate (per 100 000)		221.71	103.04	87.52	76.27	90.95	190.48	161.64		
Age-standardized rate (per 100 000)		141.27	97.84	92.88	83.64	96.36	102.26	167.38		
Age- and area-adjusted (HR1)^a^		**1.62** **(1.38–1.91)**	1.26 (0.98–1.62)	1.10 (0.97–1.25)	1.00 (Reference)	1.15 (0.99–1.33)	**1.26** **(1.03–1.55)**	**2.10** **(1.68–2.63)**	7.2% (*P* = 0.373)	0.0% (*P* = 0.759)
Multivariate-adjusted (HR2)^b^		**1.77** **(1.45–2.15)**	**1.32** **(1.02–1.70)**	1.11 (0.98–1.27)	1.00 (Reference)	1.11 (0.96–1.29)	1.15 (0.91–1.44)	**1.79** **(1.43–2.24)**	23.8% (*P* = 0.247)	0.0% (*P* = 0.790)
Multivariate-adjusted, excl. ​ early death (HR3)^c^		**1.56** **(1.26–1.91)**	1.20 (0.98–1.48)	1.11 (0.96–1.28)	1.00 (Reference)	1.11 (0.93–1.31)	1.10 (0.81–1.50)	**1.88** **(1.41–2.51)**	11.5% (*P* = 0.342)	9.7% (*P* = 0.355)

*Cerebrovascular Disease*										
Number of subjects	(*n* = 191 303)	15 027	34 289	50 450	44 316	26 341	16 066	4814		
Person-years	2 359 991	173 647	416 348	626 108	554 620	329 853	200 022	59 393		
Number of deaths	(*n* = 2251)	345	397	467	455	288	220	79		
Crude rate (per 100 000)		198.68	95.35	74.59	82.04	87.31	109.99	133.01		
Age-standardized rate (per 100 000)		137.28	92.44	78.75	89.87	91.29	117.61	134.98		
Age- and area-adjusted (HR1)^a^		**1.36** **(1.06–1.74)**	1.02 (0.86–1.20)	0.87 (0.75–1.01)	1.00 (Reference)	0.99 (0.84–1.18)	**1.26** **(1.01–1.58)**	**1.52** **(1.20–1.94)**	50.0% (*P* = 0.062)	0.0% (*P* = 0.957)
Multivariate-adjusted (HR2)^b^		**1.44** **(1.10–1.88)**	1.08 (0.91–1.28)	0.88 (0.76–1.03)	1.00 (Reference)	0.94 (0.79–1.13)	1.15 (0.93–1.41)	**1.30** **(1.02–1.65)**	55.9% (*P* = 0.034)	0.0% (*P* = 0.981)
Multivariate-adjusted, excl. ​ early death (HR3)^c^		1.32 (0.95–1.84)	1.07 (0.88–1.29)	0.88 (0.72–1.06)	1.00 (Reference)	0.95 (0.80–1.13)	1.14 (0.92–1.42)	**1.52** **(1.16–1.99)**	50.3% (*P* = 0.060)	0.0% (*P* = 0.959)

*Other Causes*										
Number of subjects	(*n* = 191 303)	15 027	34 289	50 450	44 316	26 341	16 066	4814		
Person-years	2 359 991	173 647	416 348	626 108	554 620	329 853	200 022	59 393		
Number of deaths	(*n* = 5501)	1008	1089	1264	957	641	389	153		
Crude rate (per 100 000)		580.49	261.56	201.88	172.55	194.33	194.48	257.61		
Age-standardized rate (per 100 000)		410.16	251.81	23.32	187.3	202.61	201.71	264.17		
Age- and area-adjusted (HR1)^a^		**2.27** **(1.91–2.69)**	**1.40** **(1.19–1.64)**	**1.14** **(1.00–1.29)**	1.00 (Reference)	1.10 (0.95–1.28)	1.12 (0.99–1.26)	**1.45** **(1.22–1.73)**	61.6% (*P* = 0.016)	0.0% (*P* = 0.936)
Multivariate-adjusted (HR2)^b^		**2.32** **(1.98–2.72)**	**1.44** **(1.23–1.68)**	**1.15** **(1.02–1.29)**	1.00 (Reference)	1.08 (0.94–1.24)	1.05 (0.94–1.19)	**1.31** **(1.10–1.56)**	52.7% (*P* = 0.048)	0.0% (*P* = 0.894)
Multivariate-adjusted, excl. ​ early death (HR3)^c^		**2.08** **(1.83–2.36)**	**1.39** **(1.19–1.63)**	1.14 (0.99–1.31)	1.00 (Reference)	1.05 (0.94–1.17)	1.08 (0.95–1.23)	**1.30** **(1.07–1.57)**	13.3% (*P* = 0.328)	0.0% (*P* = 0.632)

^a^Adjusted for age (years, continuous) and area (for JPHC-I, JPHC-II, and JACC only) (HR1).

^b^Further adjusted for cigarette smoking (never smoker, past smoker, or current smoker), alcohol drinking (nondrinkers [never- or ex-drinker], occasional drinkers (less than once per week), regular drinkers (almost daily for OHSAKI and 3-pref AICHI; ≥5 days/week for JPHCI, JPHCII, and JACC; ≥5 times/week for MIYAGI; and ≥4–6 days/week for TAKAYAMA), history of hypertension (no, yes), history of diabetes (no, yes), and leisure-time sports or physical exercise (less than almost daily, almost daily) (HR2).

^c^Excluding deaths within 5 years (HR3). Bold text: *P* < 0.05.

When men were stratified by smoking status, the association between mortality and low BMI was generally more pronounced among current smokers than among never smokers (Table 4). This modification effect was most pronounced in cancer mortality, for which the observed risk elevation in the low BMI range disappeared among never smokers but remained among current smokers. The HRs for BMI ranges 14 to 19, 19 to 21, and 21 to 23 kg/m^2^ were 1.05, 0.96, and 0.95, respectively, for never smokers and 1.49, 1.23, and 1.11 for current smokers. The heterogeneity in outcomes may be due in part to the relatively small sample size in the stratified analysis, and the results may not affect the above findings.

**Table 4.** Pooled analysis of BMI and mortality, stratified by smoking status (Men)

		14–<19	19–<21	21–<23	23–<25	25–<27	27–<30	30–<40	Heterogeneity I squared (%) and *P* for the	
		HR (95% CI)	HR (95% CI)	HR (95% CI)	HR (95% CI)	HR (95% CI)	HR (95% CI)	HR (95% CI)	lowest category	highest category
*All Causes*										
Never smokers, multivariate-adjusted^a^		**1.48** **(1.25–1.77)**	1.16 (0.996–1.34)	0.98 (0.89–1.08)	1.00 (Reference)	0.91 (0.80–1.04)	1.05 (0.90–1.23)	1.42 (0.99–2.02)	32.9% (*P* = 0.177)	54.8% (*P* = 0.039)
Number of subjects	(*n* = 32 227)	1479	4527	8111	9060	5481	2899	670		
Person-years	405 361	17 107	55 839	102 982	114 276	69 710	37 126	8322		
Number of deaths	(*n* = 4422)	417	784	1110	1048	589	362	112		
Crude rate (per 100 000)		2437.61	1404.04	1077.86	917.08	844.93	975.07	1345.83		
Current smokers, multivariate-adjusted^a^		**1.68** **(1.50–1.88)**	**1.22** **(1.16–1.29)**	**1.09** **(1.04–1.15)**	1.00 (Reference)	0.96 (0.90–1.03)	1.06 (0.98–1.16)	**1.40** **(1.21–1.64)**	63.5% (*P* =0.012)	0.0% (*P* = 0.780)
Number of subjects	(*n* = 85 659)	5994	17 168	24 400	20 958	10 816	5186	1137		
Person-years	1 039 570	66 679	206 925	297 395	258 013	132 793	63 968	13 797		
Number of cases	(*n* = 14 191)	1828	3353	3960	2846	1363	662	179		
Crude rate (per 100 000)		2741.49	1620.39	1331.56	1103.05	1026.41	1034.89	1297.39		

*Cancer*										
Never smokers, multivariate-adjusted^a^		1.05 (0.81–1.36)	0.96 (0.81–1.15)	0.95 (0.82–1.11)	1.00 (Reference)	0.80 (0.61–1.04)	0.97 (0.77–1.21)	1.33 (0.91–1.94)	5.5% (*P* = 0.385)	0.0% (*P* = 0.874)
Number of subjects	(*n* = 32 227)	1479	4527	8111	9060	5481	2899	670		
Person-years	389 623	16 886	54 538	100 100	106 911	67 275	35 848	8066		
Number of deaths	(*n* = 1277)	90	211	340	345	162	99	30		
Crude rate (per 100 000)		532.99	386.88	339.66	322.70	240.80	276.16	371.92		
Current smokers, multivariate-adjusted^a^		**1.49** **(1.23–1.81)**	**1.23** **(1.12–1.36)**	**1.11** **(1.02–1.20)**	1.00 (Reference)	0.97 (0.83–1.13)	0.98 (0.85–1.12)	1.30 (0.95–1.78)	68.5% (*P* = 0.004)	27.7% (*P* = 0.227)
Number of subjects	(*n* = 85 659)	5994	17 168	24 400	20 958	10 816	5186	1137		
Person-years	1 004 029	65 435	201 068	287 194	248 306	127 677	61 163	13 186		
Number of cases	(*n* = 5845)	664	1425	1701	1194	557	242	62		
Crude rate (per 100 000)		1014.74	708.72	592.28	480.86	436.26	395.67	470.19		

*Heart Disease*										
Never smokers, multivariate-adjusted^a^		1.36 (0.77–2.41)	1.11 (0.83–1.48)	0.93 (0.72–1.20)	1.00 (Reference)	1.09 (0.79–1.52)	1.35 (0.84–2.18)	**1.93** **(1.01–3.67)**	41.4% (*P* = 0.129)	13.1% (*P* = 0.331)
Number of subjects	(*n* = 32 227)	1479	4527	8111	9060	5481	2899	670		
Person-years	389 623	16 886	54 538	100 100	106 911	67 275	35 848	8066		
Number of deaths	(*n* = 515)	46	89	124	120	78	45	13		
Crude rate (per 100 000)		272.42	163.19	123.88	112.24	115.94	125.53	161.16		
Current smokers, multivariate-adjusted^a^		**1.27** **(1.03–1.56)**	0.98 (0.82–1.18)	0.99 (0.83–1.18)	1.00 (Reference)	1.10 (0.91–1.34)	1.25 (0.92–1.71)	**1.81** **(1.18–2.77)**	14.4% (*P* = 0.320)	17.1% (*P* = 0.300)
Number of subjects	(*n* = 85 659)	5994	17 168	24 400	20 958	10 816	5186	1137		
Person-years	1 004 029	65 435	201 068	287 194	248 306	127 677	61 163	13 186		
Number of cases	(*n* = 1865)	211	380	514	391	222	115	32		
Crude rate (per 100 000)		322.45	188.99	178.97	157.47	173.88	188.02	242.68		

*Cerebrovascular Disease*										
Never smokers, multivariate-adjusted^a^		1.32 (0.91–1.93)	1.32 (0.90–1.93)	0.99 (0.76–1.29)	1.00 (Reference)	1.10 (0.81–1.49)	1.23 (0.85–1.77)	**2.61** **(1.35–5.04)**	0.0% (*P* = 0.851)	41.2% (*P* = 0.147)
Number of subjects	(*n* = 32 227)	1479	4527	8111	9060	5481	2899	670		
Person-years	389 623	16 886	54 538	100 100	106 911	67 275	35 848	8066		
Number of deaths	(*n* = 493)	43	92	119	109	73	42	15		
Crude rate (per 100 000)		254.65	168.69	118.88	101.95	108.51	117.16	185.96		
Current smokers, multivariate-adjusted^a^		**1.55** **(1.28–1.87)**	1.20 (0.99–1.47)	1.02 (0.87–1.19)	1.00 (Reference)	0.98 (0.80–1.20)	1.10 (0.85–1.44)	1.41 (0.75–2.68)	0.0% (*P* = 0.611)	31.0% (*P* = 0.203)
Number of subjects	(*n* = 85 659)	5994	17 168	24 400	20 958	10 816	5186	1137		
Person-years	1 004 029	65 435	201 068	287 194	248 306	127 677	61 163	13 186		
Number of cases	(*n* = 1430)	193	341	381	288	141	70	16		
Crude rate (per 100 000)		294.95	169.59	132.66	115.99	110.43	114.45	121.34		

*Other Causes*										
Never smokers, multivariate-adjusted^a^		**1.99** **(1.53–2.59)**	**1.28** **(1.02–1.61)**	1.00 (0.79–1.28)	1.00 (Reference)	0.88 (0.72–1.09)	1.05 (0.81–1.37)	1.02 (0.65–1.60)	32.7% (*P* = 0.178)	0.0% (*P* = 0.554)
Number of subjects	(*n* = 32 227)	1479	4527	8111	9060	5481	2899	670		
Person-years	389 623	16 886	54 538	100 100	106 911	67 275	35 848	8066		
Number of deaths	(*n* = 1434)	187	288	357	317	163	101	21		
Crude rate (per 100 000)		1107.43	528.07	356.64	296.51	242.29	281.74	260.34		
Current smokers, multivariate-adjusted^a^		**2.24** **(1.93–2.60)**	**1.35** **(1.23–1.49)**	**1.16** **(1.03–1.30)**	1.00 (Reference)	0.93 (0.80–1.08)	1.10 (0.94–1.28)	**1.51** **(1.06–2.15)**	40.5% (*P* = 0.121)	33.8% (*P* = 0.170)
Number of subjects	(*n* = 85 659)	5994	17 168	24 400	20 958	10 816	5186	1137		
Person-years	1 004 029	65 435	201 068	287 194	248 306	127 677	61 163	13 186		
Number of cases	(*n* = 4627)	738	1120	1245	863	397	208	56		
Crude rate (per 100 000)		1127.83	557.03	433.51	347.55	310.94	340.08	424.69		

^a^Adjusted for age (years, continuous) and area (for JPHC-I, JPHC-II, and JACC only), cigarette smoking (never smoker, past smoker, current smoker of 1–19 cigarettes/day or ≥20 cigarettes/day), alcohol drinking (nondrinkers [never- or ex-drinker], occasional drinkers (less than once per week), regular drinkers (almost daily for OHSAKI and 3-pref AICHI; ≥5 days/week for JPHCI, JPHCII, and JACC; ≥5 times/week for MIYAGI; and ≥4–6 days/week for TAKAYAMA), history of hypertension (no, yes), history of diabetes (no, yes), and leisure-time sports or physical exercise (less than almost daily, almost daily).

The data suggest that approximately 0.9% and 1.5% of total deaths were attributable to a high BMI (≥27 kg/m^2^) in men and women, respectively, as were 0.2% and 1.0% of cancer deaths, 2.8% and 2.7% of heart disease deaths, and 1.5% and 1.9% of cerebrovascular deaths.

## DISCUSSION

In this pooled analysis of more than 350 000 Japanese, an elevated risk of all-cause mortality for both high and low BMI levels was observed in both sexes. This association remained after excluding early deaths during follow-up and after restricting the analysis to never smokers (in men). The results conform with most previous cohort studies in Japan, which showed a U-shaped^7^^,^^9^ or reverse J-shaped association.^10^ Other studies showed no obvious increase in risk due to obesity in men^5^^,^^8^ or women,^6^ due to the older age of the subjects or the small number of subjects in the respective categories. All-cause mortality was lowest at a BMI range of 23 to 27 kg/m^2^ in men and 21 to 27 kg/m^2^ in women. Above this range, a significant increase in risk was observed only at a BMI range of 30 to 40 kg/m^2^ in men and 27 kg/m^2^ or higher in women. Men with a BMI of 27 to 30 kg/m^2^ had a slightly elevated risk, which was not statistically significant. Four of 7 individual studies included in the pooled analysis showed an elevated risk, and among these, 3 found a statistically significant association; the HR range was 1.13 to 1.36. Therefore, we believe that a BMI greater than 27 kg/m^2^ should be defined as a high-risk group for overall mortality in both men and women and that it is not necessary to set a higher or lower cut-off point in this population.

Cancer accounted for 37% (39% in men and 35% in women) of overall deaths. The association of BMI with cancer was similar to that observed for BMI and all-cause mortality in men. It has been observed in many studies that low BMI is associated with increased risk of cancer.^21^^,^^29^^,^^30^ As the effect-measure modification by cigarette smoking suggests, the risk elevation with low BMI in men is probably mostly due to smoking-related cancers (eg, cancers of the lung and esophagus, among others). In this population, most women were nonsmokers and thus no risk elevation was observed among women with a low BMI. Evidence of a positive association between high BMI and cancer risk comes mainly from Western populations, as shown in the Cancer Prevention Study-II^31^^–^^34^ and the Million Women Study.^35^^,^^36^ Among previous cohort studies conducted in Japan, only 1 showed a statistically significant positive association between high BMI and cancer incidence in women, which was attributed to cancers of the breast (postmenopausal), endometrium, gallbladder, and colorectum.^37^ That study and another study^21^ suggested that men were also at increased risk, and another study found that both men and women were at increased risk.^30^ However, none of these findings were statistically significant. This may be due to the smaller proportion of overweight people in Japan as compared with Western countries. By pooling data, the present study revealed that obesity does increase the risk of mortality from cancer, although the contribution to the overall cancer burden was small.

For heart disease and cerebrovascular disease, a U- or J-shaped association was observed among men and women. Many epidemiologic studies have shown that obesity is a significant risk factor for developing heart disease and cerebrovascular disease. A continuous positive association was observed between BMI and the incidences of ischemic heart disease and stroke^38^ and mortality^29^ in collaborative analyses of prospective studies involving 310 000 participants from the Asia-Pacific region and 900 000 participants mainly from Western Europe and North America, respectively. In particular, dyslipidemia, diabetes mellitus, and hypertension are positively related to obesity.^39^^–^^41^ These intermediate factors related to the disease may be largely accounted for by the elevated risk associated with a high BMI. However, the elevated risk was still significant even after controlling for histories of diabetes and hypertension (HR2). This suggests that another mechanism not explained by these factors might exist within the pathway. Funada et al and Cui et al reported an elevated risk of ischemic heart disease and hemorrhagic stroke not only among individuals with a high BMI, but also among those with a low BMI.^27^^,^^28^ Several studies identified an association between low serum cholesterol level and hemorrhagic stroke.^42^^,^^43^ Serum cholesterol level is positively correlated with BMI, which might explain the finding of elevated risk of hemorrhagic stroke among those with a low BMI. However, a definitive interpretation is not possible and further studies of the causal mechanisms linking low cholesterol and hemorrhagic stroke are needed.^43^ In addition to cigarette smoking and preexisting disease, suggested mechanisms for the observed elevated risk of heart disease and cerebrovascular disease among individuals with low BMI include several cardiovascular abnormalities, such as reduced ventricular mass, valvular dysfunction, electrocardiographic changes, cardiac myofibril damage, and compromised immunity.^28^

As was the case for cause-specific mortality and all-cause mortality, both high and low BMI values were related to excess risk of other-cause mortality. Although the specific causes of death are unknown, some interpretations are possible. As mentioned above, a high BMI is associated with an increased risk of major chronic diseases and more people are likely to die from the complications of such diseases. Elevated risk was also observed among those with a low BMI, which suggests that people with a low BMI have less resistance to various diseases, including infectious, respiratory, or inflammatory diseases.

In Western countries, more attention is paid to overweight and obesity than to low BMI. In a collaborative analysis of data from 57 prospective studies of almost 900 000 adults, mostly in Western Europe and North America, a U-shaped association, similar to ours, was observed for overall mortality, with the lowest risk at a BMI of 22.5 to 25 kg/m^2^ after controlling for early follow-up and smoking status.^29^ However, the PAF was calculated for higher BMIs only, which seemed to be largely causal. Based on the relative risks and recent population BMI values, approximately 29% of vascular deaths and 8% of neoplastic deaths in late middle age in the United States were attributable to having a BMI greater than 25 kg/m^2^. In the United Kingdom, the corresponding proportions were approximately 23% and 6%. In France, a working group of the International Agency for Research on Cancer reported that the PAF of all-cancer mortality due to obesity and overweight—calculated by summing the results of obesity-related cancers (ie, esophageal [adenocarcinoma], colorectal, kidney, corpus uteri, and breast [in postmenopausal women] cancers)—was 1.1% for men and 2.3% for women.^44^

The elevated risk of mortality among those within the low BMI range was most apparent for diseases of other causes, whose past history was not deleted. This indicates that reverse causation, namely, bias caused by preexisting illness and attendant weight loss, might partially explain the observed findings. To eliminate this possibility, we excluded deaths within 5 years, the method most frequently proposed to control for possible illness-related weight loss (IRWL).^23^ We found that most RRs were attenuated and that heterogeneity across studies improved in the low BMI range. In the high BMI range, some RRs were attenuated while others were not, CIs increased, and heterogeneity was unchanged or increased. Using this indirect approach, individuals with IRWL are not necessarily excluded and those who are excluded do not necessarily have IRWL, which could introduce new sources of bias. Because no adequate method has been established to control for the effect of reverse causation, it is not possible to totally eliminate or clearly reveal the magnitude of the effect. However, the high prevalence of lean people in Japan indicates that a low BMI might be associated with mortality risk. In a pooled analysis of more than 1 million Asians, Zheng et al observed that underweight was associated with a substantially increased risk of death in all Asian populations.^4^ They indicated that inadequate or incomplete control of confounding or reverse-causation bias might, in part, explain this increased risk. As Flegal et al indicate in their recent study, there is a need for studies with a more restricted focus and greater detail. Such studies might consider weight change or develop new methods of causal modeling.^45^

This study has several limitations. First, measures of abdominal obesity, such as waist circumference and waist-to-hip ratio, were not available. In the European Prospective Investigation on Cancer prospective study, both waist circumference and waist-to-hip ratio were strongly associated with risk of death, independent of BMI.^46^ Therefore, the number of deaths attributable to all adiposity-related factors is probably greater than the present estimates. Second, the present BMI calculation was based on self-reported values. To minimize the effect of unreliable reporting, we excluded individuals reporting a BMI less than 14 or 40 kg/m^2^ or higher. In the Takayama Study, the intraclass correlation coefficients between self-reported and measured height and weight in a subsample were 0.93 and 0.97 in both sexes, respectively.^18^ In the JPHC study (combined JPHC-I and II, corresponding to 31.3% of the pooled dataset), self-reported BMI was slightly lower than measured BMI. In comparing self-reported height and weight with available data from health check-ups (11 274 men and 21 196 women), the Spearman correlation coefficient was 0.89 and 0.90 for men and women, respectively.^21^ Similar underestimates of BMI, especially at higher weights, were also observed in a Western population.^47^ It is uncertain whether the same was true for the other 4 studies; however, excess risk was observed only for a BMI of 30 kg/m^2^ or higher across most of the end points, and the abovementioned effect is not likely to be large. Third, we used only single-point measurements of BMI as an exposure and did not capture weight change during the period. Accumulating evidence suggests that both weight gain and loss in adult life are associated with increased risk of mortality. We have previously observed that mortality from all causes and cancer is elevated by a weight loss of 5 kg or more after age 20 years^48^ and during middle age,^49^ whereas mortality from cardiovascular disease is elevated by a weight loss of 5 kg or more after age 20 in men^48^ and weight gain during middle age in women.^49^ Our combined findings indicate that maintaining an adequate weight in adulthood may be an important strategy for improving mortality in Japan. Limitations might also exist due to the process used for handling missing values. We chose to create an indicator term for missing data for each covariate, which might have led to biased estimates of the overall effect of the study exposure.^50^

The strength of this study is that it included most of the ongoing prospective studies in Japan, with overlapping birth generations and a similar survey time period. Therefore, pooling of these studies allows for a stable quantitative estimate of the impact of relative weight among Japanese. In addition, the categories of BMI and covariates used were identical among studies, which removes a potential source of heterogeneity that can occur in a meta-analysis of published literature.

In summary, the lowest risks of total mortality and mortality from major causes of diseases were observed at a BMI of 23 to 27 kg/m^2^ for men and 21 to 27 kg/m^2^ for women in middle-aged and elderly Japanese. Because there was no elevation of risk for a BMI of 21 to 23 in never-smoking men, we conclude that a BMI of 21 to 27 kg/m^2^ is associated with the lowest mortality risk in both sexes.
